# Case Report of a Left-sided Superior Vena Cava Causing Unique Positioning of Central Line

**DOI:** 10.5811/cpcem.2020.8.48372

**Published:** 2020-10-09

**Authors:** Michael Mancera, Nicholas Genthe, Nicholas Lepa

**Affiliations:** * University of Wisconsin, BerbeeWalsh Department of Emergency Medicine, Madison, Wisconsin; † University of Wisconsin School of Medicine and Public Health, Madison, Wisconsin

**Keywords:** Left sided superior vena cava, congenital venous malformation, malpositioned central venous catheter

## Abstract

**Introduction:**

Persistent left-sided superior vena cava is a rare congenital venous malformation. While often clinically asymptomatic, these variations in normal anatomy may give rise to complications with central venous catheter placement.

**Case Report:**

We present a case of a 71-year-old male who presented to the emergency department with sepsis of unknown etiology. A right-sided central venous catheter was placed, and due to a persistent left-sided superior vena cava the post-procedure chest radiograph showed a uniquely positioned catheter tip within the left atrium.

**Conclusion:**

A persistent left-sided superior vena cava may lead to uniquely positioned catheter tip placement on post-procedural imaging. This case demonstrates the need to consider variants in normal venous anatomy, such as persistent left-sided superior vena cava, to aid with correct interpretation of post-procedure imaging findings.

## INTRODUCTION

A persistent, left-sided superior vena cava (SVC) is a congenital venous anomaly in the chest that results when the left anterior cardinal vein is not obliterated during normal fetal development. The incidence of a left-sided SVC is 0.3–0.5% in the general population, and increases to 4.5% in individuals with congenital heart defects.^1^ Of patients with a left-sided SVC, 90% will have an accompanying right-sided SVC, termed SVC duplication.^2^ A majority of patients with a left-sided SVC are asymptomatic, although a subset of patients may present with cyanosis, secondary to right-to-left shunting, as well as rhythm abnormalities and conduction disturbances.^3^ The vast majority of left-sided SVC cases are found incidentally on cross-sectional imaging of the chest.^4^ Our case highlights the challenges of performing central venous catheterization on patients with persistent left-sided SVC and emphasizes significant imaging findings for these patients.

## CASE REPORT

Our patient was a 71-year-old male with a past medical history of coronary artery disease, diabetes, hypertension, hyperlipidemia, benign prostatic hyperplasia, and obesity who presented to the emergency department (ED) with headaches and confusion. He was a poor historian secondary to altered mental status; therefore, a limited history was obtained. His wife noted that he had been acting confused over the prior 24 hours. The patient also endorsed some neck stiffness, which resolved upon presentation to the ED. No history of trauma was reported.

**Image 1:**
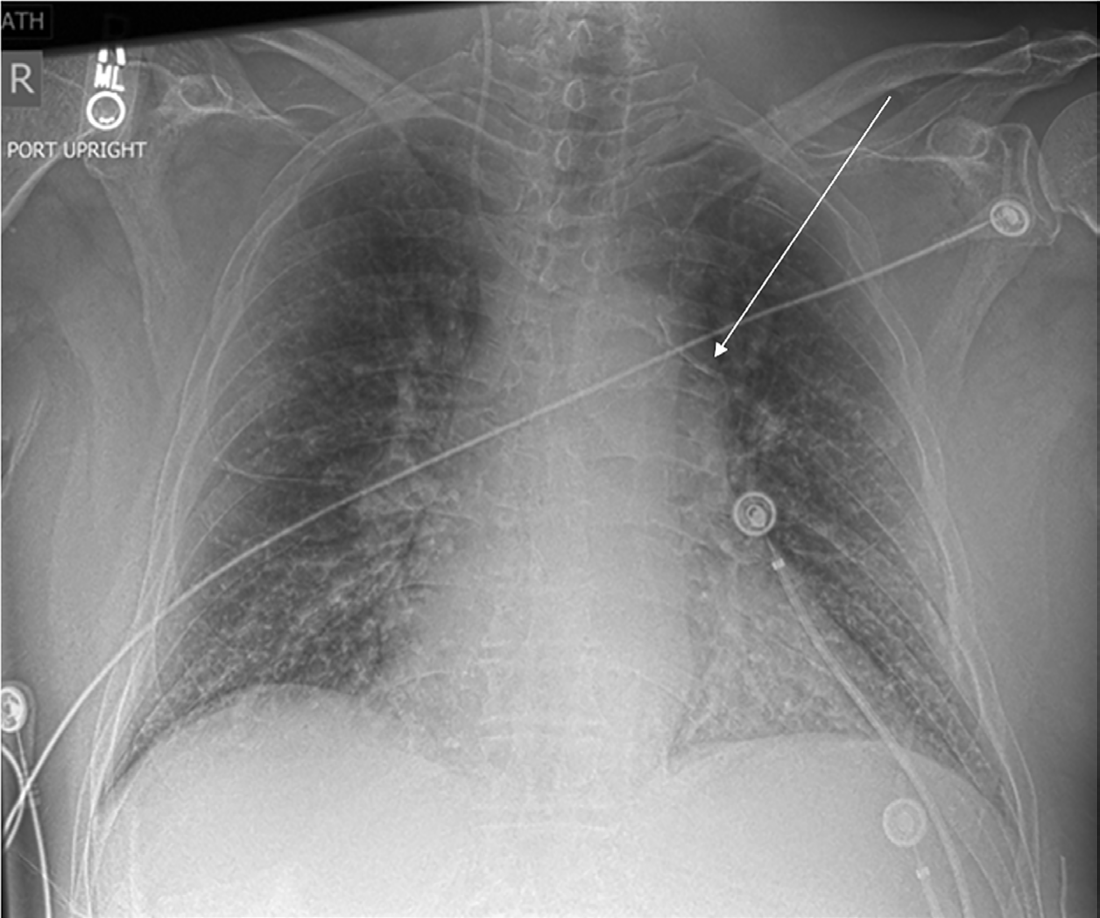
Chest radiograph obtained post right-sided internal jugular vein catheter placement, which shows catheter crossing midline right to left terminating within the left atrium (arrow).

His vitals upon presentation to the ED included an oral temperature of 36.1º Celsius, blood pressure of 62/40 millimeters mercury, heart rate of 107 beats per minute, respiratory rate of 18 breaths per minute, and oxygen saturation of 98% on room air. Initial laboratory values for this patient included an elevated white blood cell count to 14.3 K/units per liter (uL) (reference [ref] range 3.8–10. /uL), and a lactate of 5.4 millimoles (mmol) /L (ref <2.0mmol/L). The patient was suspected to have septic shock from an infection of unknown etiology. Due to continued hypotension despite intravenous fluid resuscitation, central venous access was obtained by the emergency physicians for vasopressor administration. A central venous catheter (CVC) was placed within the right internal jugular (IJ) vein under ultrasound guidance. The patient tolerated this procedure without complication, and a chest radiograph was obtained following the procedure to confirm proper line placement (Image 1). At this time, the catheter was noted to be abnormally positioned, as it crossed midline from right-to-left, terminating in the left atrium.

**Image 2:**
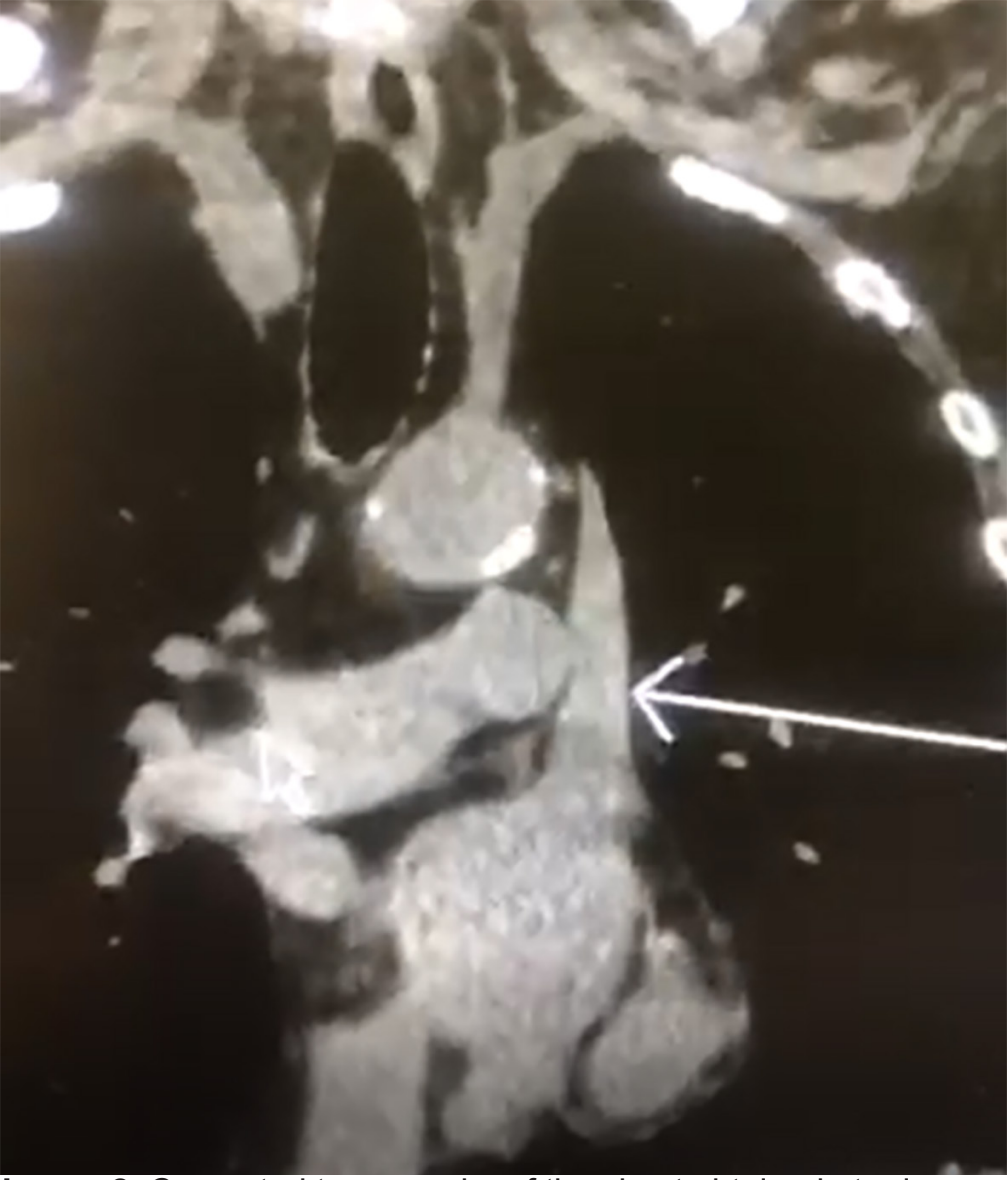
Computed tomography of the chest obtained at prior hospitalization demonstrates the presence of a persistent left-sided superior vena cava (arrow), as well as the complete absence of an accompanying right-sided superior vena cava.

Upon further chart review, including prior imaging, the patient was found to have an existing persistent left-sided SVC draining into the left atrium, based on a prior computed tomography of the chest (Image 2). There was no evidence of a right-sided superior vena cava on the prior advanced imaging. The patient was ultimately diagnosed with ascending cholangitis, which was successfully managed with biliary drainage. He did have several other complications throughout his hospital course secondary to his other medical comorbidities, all of which were unrelated to his SVC.

## DISCUSSION

Left-sided SVC is the most common congenital venous anomaly in the chest and is known to have an increased prevalence in patients with congenital cardiac abnormalities. The embryologic origin of persistent left-sided SVC stems from failure of obliteration of the left anterior cardinal vein during normal fetal development.^5^ This results in significant variability in anatomy with a majority of left-sided SVC patients draining into the coronary sinus, and a minority draining into the left atrium or elsewhere. While an overwhelming majority (>90%) of these patients are asymptomatic, these anatomical variations become clinically relevant in several scenarios. Around 10% of patients with a persistent left-sided SVC will have drainage into the left atrium, leading to a right to left shunt. This shunting allows for the mixing of deoxygenated blood from the venous circuit with oxygenated blood from the left atrium resulting in cyanosis, as well as an increased embolic risk.^6^

CPC-EM Capsule

What do we already know about this clinical entity?

*A persistent left-sided superior vena cava is a rare congenital venous malformation that may complicate central venous catheter placement*.

What makes this presentation of disease reportable?

*This case shows imaging of a malpositioned, right-sided central venous catheter tip secondary to a persistent left-sided superior vena cava with no right-sided superior vena cava*.

What is the major learning point?

*Consider variants in venous anatomy to aid in the correct interpretation of post-procedure imaging after central line placement*.

How might this improve emergency medicine practice?

*Early identification of variations in venous anatomy may prevent work-up for additional causes of a malpositioned catheter tip on imaging*.

Patients with persistent left-sided SVC are also at an increased risk for anomalies of the cardiac conduction system as well as congenital heart defects. This leads to increased incidence of supraventricular tachycardia, atrial fibrillation, atrial flutter, Wolff-Parkinson-White syndrome, and atrioventricular conduction blocks, as well as atrial septal defects, ventricular septal defects, and conotruncal defects.^7^,^8^ It is for this reason that when these patients are identified it is recommended that they undergo thorough cardiac workup, including electrophysiological studies, echocardiogram, and annual clinical follow-up.

It is important for emergency physicians to understand these variants of normal venous anatomy as they are often diagnosed incidentally on cardiac imaging, including echocardiography. As emergency physicians frequently use point-of-care ultrasound, it is increasingly likely that they may encounter a case of persistent left-sided SVC on transthoracic echocardiogram. Characteristic findings on point-of-care echocardiogram include a dilated coronary sinus without evidence of elevated, right-sided filling pressures. When this diagnosis is suspected it should be confirmed with a saline contrast echocardiography microbubble study or additional vascular imaging studies.^9^ The main ramification of a diagnosis of persistent left-sided SVC is the increased potential for congenital heart defects and dysrhythmias as noted above. When identified this should prompt the evaluation for additional cardiac malformations. The presence of associated cardiac abnormalities is ultimately the prognostic indicator that drives mortality in these patients.^10^ The most appropriate setting for further workup is determined by the patient’s clinical status; however, most cases are asymptomatic and may be followed on an outpatient basis with cardiology.

A knowledge of anatomical variations within the venous system such as persistent left-sided SVC is critically important for clinicians who perform emergent procedures such as central venous line placement. Proper placement of a CVC requires that the tip of the catheter be placed in as large a central vein as possible and parallel with the long axis of the vein to avoid abutting the catheter against the vein or heart wall.^11^ It is estimated that around 5% of CVC placements result in a malpositioned catheter.^12^ The presence of a persistent left-sided SVC is an important risk factor for improper positioning of CVCs.^13^ In these cases, catheter placement into the coronary sinus is an understood complication of left-sided CVC placement, resulting in a CVC with an abnormal, left paramediastinal intrathoracic course noted on radiograph.^13^ It is also noted that many of these patients with CVC placement go unnoticed if a right-sided attempt is made. There are no known publications documenting radiographic imaging findings suggestive of an incorrectly positioned right-sided CVC crossing the midline of the chest as a result of a persistent left-sided SVC.

In this case the patient presented with sepsis from an unknown source and needed emergent central line placement for vasopressor administration to maintain adequate perfusion pressures. It was unknown to the clinical team at the time that this patient not only had a persistent left-sided SVC, which drained into the left atrium, but also had the complete absence of a right-sided SVC. This combination is exceedingly rare and explains the post-procedure imaging findings of a right-sided IJ vein CVC crossing the patient’s midline. Given that this patient had the complete absence of a right-sided SVC, the catheter tip was tracking through the left brachiocephalic vein. At this point the left brachiocephalic vein merges with a persistent left-sided SVC draining directly into the left atrium. This is where the catheter tip eventually terminated.

Notably, most patients with a persistent left-sided SVC have an associated right-sided SVC that drains into the right atrium. In those cases, right-sided IJ vein placement would not be affected as the right-sided venous circulation is appropriately developed. However, in this case, given the anatomy of the persistent left-sided SVC without an associated right-sided SVC, the catheter crossed the midline and terminated in the SVC as shown in the chest radiograph. It is also important to note that had a left-sided IJ been attempted, the catheter tip would have continued into the left atrium instead of crossing into the right atrium as expected given the complete absence of a right-sided SVC draining into the right atrium.

Based on the post-procedure imaging findings shown, it would be reasonable to conclude that the abnormal positioning could represent arterial line placement, with the CVC entering the aorta and then crossing the midline of the chest. This complication would represent a vascular emergency. However, our case highlights the importance of considering other etiologies for the central line crossing the midline, such as persistent left-sided SVC.

## CONCLUSION

This case illustrates an unexpected central venous catheter position on chest radiograph after successful placement of a right-sided internal jugular venous catheter, due to the congenital malformation of a persistent left-sided superior vena cava. This could easily be mistaken for a vascular complication such as arterial line placement. Persistent left-sided SVC is a clinically important anatomical variant of the central venous system. Emergency physicians should be aware of this variant when placing central venous catheters as it may result in a CVC catheter tip appearing malpositioned on post-procedure imaging. A greater understanding of anatomic variants can aid with correct interpretation of emergent imaging findings. It is, therefore, important for emergency physicians to consider these variants of normal anatomy prior to placing a CVC.

## References

[1] Tyrak KW, Holda J, Holda MK (2017). Persistent left superior vena cava. Cardiovasc J Afr.

[2] Ruano CA, Marinho-da-Silva A, Donato P (2015). Congenital thoracic venous anomalies in adults: morphologic MR imaging. Curr Probl Diagn Radiol.

[3] Goyal SK, Punnam SR, Verma G (2008). Persistent left superior vena cava: a case report and review of literature. Cardiovasc Ultrasound.

[4] Kellman GM, Alpern MB, Sandler MA (1988). Computed tomography of vena caval anomalies with embryologic correlation. Radiographics.

[5] Huang SK (1986). Persistent left superior vena cava in a man with ventricular fibrillation. Chest.

[6] Morgan LG, Gardner J, Calkins J (2015). The incidental finding of a persistent left superior vena cava: implications for primary care providers-case and review. Case Rep Med.

[7] Duymus M, Yesilkaya Y, Orman G (2012). Persistent left superior vena cava draining to the left atrium: a case report and review of the literature. Pol J Radiol.

[8] Mark JB, Slaughter TF, Gerald Reves J, Miller RD (2009). Cardiovascular monitoring. Anesthesia.

[9] Kim H, Kim JH, Lee H (2014). Persistent left superior vena cava: diagnosed by bedside echocardiography in a liver transplant patient: a case report. Korean J Anesthesiol.

[10] Galindo A, Gutiérrez-Larraya F, Escribano D (2007). Clinical significance of persistent left superior vena cava diagnosed in fetal life. Ultrasound Obstet Gynecol.

[11] Pikwer A, Bååth L, Davidson B (2008). The incidence and risk of central venous catheter malpositioning: a prospective cohort study in 1619 patients. Anaesth Intensive Care.

[12] Gibson F, Bodenham A (2013). Misplaced central venous catheters: applied anatomy and practical management. Br J Anaesth.

[13] Wang L, Liu ZS, Wang CA (2016). Malposition of central venous catheter: presentation and management. Chin Med J (Engl).

